# C-reactive protein for detection of colorectal anastomotic leakage and safer early discharge

**DOI:** 10.1590/0100-6991e-20253798-en

**Published:** 2025-09-23

**Authors:** TOMAS MDM MARQUES, ELIEL M ROVARIS, REBECA H NAHIME, PAULO ROBERTO STEVANATO, BRUNA EC KUPPER, ANDRE L BETTIATI, TIAGO S BEZERRA, RENATA M TAKAHASHI, WILSON T NAKAGAWA, ADEMAR LOPES, SAMUEL AGUIAR

**Affiliations:** 1 - A.C. Camargo Cancer Center, Centro de Referência de Tumores Colorretais - São Paulo - SP - Brasil

**Keywords:** Anastomotic Leak, Colorectal Surgery, C-Reactive Protein, Enhanced Recovery After Surgery, Fístula anastomótica, Cirurgia Colorretal, Proteína C-Reativa, Aprimoramento de recuperação cirúrgica

## Abstract

**Introduction:**

Colorectal anastomotic leakage is associated with increased mortality, need for reoperation, diverting stomas, delayed discharge and higher costs. Early detection of anastomotic leaks can significantly impact morbidity and clinical outcomes. The aim of this study is to identify the role of C-reactive protein (CRP) in the early detection of anastomotic leakage to improve treatment and fasten rehabilitation.

**Methods:**

This is a prospective study that included 180 patients submitted to colectomies at AC Camargo Cancer Center from October 2021 to December 2022. C-reactive protein was systematically assessed on the third day after surgery. Postoperative care was provided by a single colorectal team based on our protocol for early recovery, which includes early feeding and mobilization, opioid avoidance and antibiotic prophylaxis.

**Results:**

180 patients were included in the study, comprising 79 women and 101 men, with a mean age of 62 years. Eighteen out of 180 patients presented with anastomotic leakage (10%). Analyzing the ROC curve for CRP, the area under the curve (AUC) was 0.860. The cutoff value was 11.2mg/dL, with an ROC of 0.81, resulting in a sensitivity of 77.8% and specificity of 84.0%. The negative predictive value (NPV) was 97.1%. An alternative cutoff with higher accuracy was 15.0mg/dL which has NPV of 96.1%, and more clinical applicability.

**Conclusion:**

A postoperative CRP of less than 15.0mg/dL, measured on the 3rd postoperative day, was effective in predicting a low risk of anastomotic leakage on asymptomatic patients allowing safe early discharge.

## RESUMO

**Objetivo:**

A fístula de anastomose colorretal está associada a maior mortalidade, necessidade de reoperações, formação de estomas de derivação, alta hospitalar tardia e aumento de custos. A detecção precoce é fundamental para reduzir morbidade e melhorar os desfechos clínicos. Este estudo teve como objetivo avaliar o papel da proteína C-reativa (PCR) na detecção precoce de fístulas anastomóticas para otimizar o tratamento e acelerar a recuperação.

**Métodos:**

Trata-se de um estudo observacional prospectivo que incluiu pacientes submetidos a colectomias no AC Camargo Cancer Center entre outubro de 2021 e dezembro de 2022. A PCR foi sistematicamente medida no 3º dia pós-operatório. O cuidado pós-operatório seguiu um protocolo institucional de recuperação precoce, incluindo alimentação e mobilização precoces, ausência de opioides e profilaxia antibiótica.

**Resultados:**

Foram analisados 180 pacientes, dos quais 101 eram homens, com idade média de 62 anos. Dezoito pacientes (10%) apresentaram fístula anastomótica. A análise da curva ROC para PCR mostrou uma área sob a curva (AUC) de 0,860. O valor de corte de 11,2mg/dL apresentou sensibilidade de 77,8% e especificidade de 84,0%, com valor preditivo negativo (VPN) de 97,1%. Um valor de corte alternativo de 15,0mg/dL mostrou maior aplicabilidade clínica, com VPN de 96,1%.

**Conclusão:**

Valores de PCR abaixo de 15,0mg/dL no 3º dia pós-operatório foram eficazes para prever baixo risco de fístula anastomótica em pacientes assintomáticos, possibilitando alta hospitalar precoce e segura.

## INTRODUCTION

Colorectal cancer is the third most common type of neoplasm among men and women in Brazil and the second most frequent cause of cancer-related death. Early diagnosis is essential to reduce the morbidity associated with treatment and mortality caused by the disease. Surgery remains the only curative treatment, although it is not free of complications^1^ ^,^ ^2^.

The most critical complication associated with colorectal surgery is anastomotic dehiscence, which occurs in 2% to 20% of cases. It is defined as any failure in the anastomosis integrity, resulting in communication between the inside of the intestinal lumen and the outside, with consequent leakage of intestinal contents.

Anastomotic dehiscence, also called fistula, increases morbidity and requires reoperation in about 85% of cases. It is an independent prognostic factor for increased local recurrence and reduced survival. A bypass ostomy is required in 50% of cases, prolonging length of hospital stay and raising health care costs. In addition, early diagnosis allows for rapid treatment and helps prevent further complications and morbidity^3^ ^-^ ^5^. 

Postoperative care involves a comprehensive evaluation that includes history, physical examination, and laboratory analysis. Symptoms of fistula are similar to those of sepsis, including tachycardia, abdominal pain, and decreased appetite, while physical examination may reveal signs of peritonitis. Late diagnosis is associated with adverse outcomes, with mortality rates that can reach 18%^6^ ^,^ ^7^. 

Several methods are used for the early diagnosis of dehiscence, such as radiological examinations, cytokines, and inflammatory markers. C-reactive protein (CRP) is an acute-phase protein used as a reliable marker of the systemic inflammatory response. It has high sensitivity in detecting infections and surgical complications^8^ ^-^ ^12^. 

To address fistula, a critical surgical complication involving morbidity and mortality, resulting in increased costs and length of hospital stay, it is imperative to explore and implement methods for early detection of this condition. This study aimed to investigate the role of C-reactive protein in the early identification of anastomotic dehiscence, through measurement on the third postoperative day of colorectal surgery, to predict complications and allow safe and early discharge.

## METHODS

We conducted a prospective cohort study involving patients diagnosed with colorectal cancer, aged over 18 years, treated with colectomy and primary anastomosis at the AC Camargo Cancer Center between October 2021 and December 2022. We measured C-reactive protein on the third day after the surgical procedure. We excluded patients undergoing emergency surgery, those without a diagnosis of colorectal cancer, individuals over 80 years of age, and those without biomarkers recorded on the third postoperative day.

Patients followed a standard institutional protocol of early recovery, consisting of the introduction of diet in the immediate postoperative period, ambulation on the same day of surgery, removal of the urinary catheter until the morning of the 1st postoperative day, avoidance of abdominal drains and gastric catheters, in addition to restriction of the use of opioids, with the objective of safe discharge on the 3rd postoperative day. To consider discharge safe, we used the following criteria: 1. Good diet acceptance; 2. Adequate intestinal transit (evacuation or elimination of flatus); 3. Absence of clinical signs of complication, defined by abdominal pain, abdominal distension, fever, tachycardia, or change in the appearance of drain contents. 

A single colorectal surgery team, with more than ten years of experience in the field, performed all surgical procedures and postoperative follow-up. We measured C-reactive protein on the morning of third postoperative day, and the surgical team monitored the daily clinical evolution. CRP was collected by intravenous puncture, and evaluated by turbidimetry in blood plasma, with a normal value defined as <0.3mg/dL.

To characterize the study population, we collected data on oncological staging, functional performance according to the Eastern Cooperative Oncology Group (ECOG), neoadjuvant therapy, previous comorbidities, such as hypertension, diabetes, and obesity, and habits, such as smoking and alcoholism. We defined anastomotic dehiscence by imaging tests that showed pneumoperitoneum, excess free fluid in the abdominal cavity, failure in the anastomosis staple line, presence of fecaloid contents in the drain, or intraoperative confirmation.

This study was previously evaluated and approved by the Ethics in Research Committee of the Antônio Prudente Foundation, under protocol number 4,908,028.

### Statistical analysis

We performed a descriptive analysis, presenting absolute and relative frequencies for qualitative variables, as well as measures such as mean, standard deviation, median, minimum and maximum values for quantitative variables.

To define a new parameter based on CRP values, we use the Receiver Operating Characteristic (ROC) Curve analysis, in which we evaluate the point that maximizes sensitivity and specificity. To evaluate this new parameter, we calculated diagnostic measures, including sensitivity, specificity, positive predictive value (PPV), negative predictive value (NPV), and accuracy.

We adopted a significance level of 5%, and statistical analyses were performed using SPSS software, version 28.

## RESULTS

We included 180 patients in this study, 79 women and 101 men, with a mean age of 62 years. We observed functional performance by the ECOG classification of 1-2 in 175 patients, 90% of whom had at least one comorbidity. Early clinical stages (CE I-II) accounted for 86.7% of cases, with 11.1% of patients receiving neoadjuvant treatment.

The most common surgical procedure was right colectomy (43 patients, 23.9%), followed by upper anterior rectum resection and sigmoidectomy (38 and 36 patients, 21.1% and 20%, respectively). Most surgeries were performed using minimally invasive techniques (87.8%). The mean length of hospital stay was five days. Of the 180 patients, 18 had anastomotic dehiscence (10%) as a complication (Table 1).

**Table 1:** Demographic characteristics of patients.

Variable	n	%
Sex		
Male	101	56.10%
Female	79	43.90%
Age		
< 49 years old	23	12.80%
50 to 75 years old	132	73.30%
> 76 years old	25	13.90%
Tumor location		
Ceccum and ascending colon	47	26.10%
Transverse colon	20	11.10%
Descending colon	15	8.30%
Sigmoid and high rectus	75	41.70%
Low rectum	23	12.80%
Staging		
Early	156	86.70%
Locally Advanced (cT4)	9	5.00%
Metastatic	15	8.30%
Neoadjuvant Therapy		
Yes	20	11.1%
No	160	88.9%
Comorbidities		
Hypertension		
Yes	86	47.8%
No	94	52.2%
Diabetes mellitus		
Yes	46	74.40%
No	134	25.60%
Obesity		
Yes	42	23.30%
No	138	76.70%
Smoking		
Yes	6	3.30%
No	174	96.70%
Alcoholism		
Yes	6	3.30%
No	174	96.70%
Variable	n	%
ECOG		
0	121	67.20%
1	57	31.70%
2	1	0.60%
3	1	0.60%
Type of surgery		
Right colectomy	43	23.90%
Enlarged right colectomy	19	10.60%
Left colectomy	15	8.30%
Sigmoidectomy	36	20.00%
Resection of upper rectum	38	21.10%
Low anterior resection with TME	23	12.80%
Total colectomy	6	3.30%
Surgical approach		
Laparoscopic	138	76.70%
Robotics	20	11.10%
Open	22	12.20%
Reoperation		
Yes	14	7.70%
No	166	92.20%
Anastomosis technique		
Manual	26	14.40%
With stapler	154	85.60%
Anastomosis dehiscence		
Yes	18	10%
No	162	90%

Of the 180 colorectal surgeries performed, 18 had fistulas; only four of these patients had CRP levels below 11.2mg/dL, and six had CRP levels below 15mg/dL. All 18 patients diagnosed with fistula received antibiotic treatment. Four already had a previous protective ileostomy, while 14 underwent a new surgical procedure. Of these, 12 required an ostomy and two underwent reanastomosis (Table 2).

**Table 2:** Characteristics of patients with fistula.

Patient	CRP on day 3 (mg/dL)	Clinical Symptoms	Surgical Procedures	Readmission	Length of hospital stay
1	11.38	Tachycardia + abdominal distension	Yes (EL - Protective ileostomy)	No	17 days
2	4.26	Abdominal pain + nausea	Yes (EL - Protective ileostomy)	Yes (11th POD)	17 days
3	5.62	Tachycardia + nausea	Yes (EL - Protective ileostomy)	No	12 days
4	9.67	Tachycardia + nausea + abdominal pain	Yes (ileodescendant anastomosis reconstruction)	No	16 days
5	11.44	Asymptomatic (8mm fistula after seeing in rectosigmoidoscopy 27 days after RTS (protective ileostomy in 01 procedure)	No	No	3 days
6	4.09	Abdominal pain + tachycardia + fever	Tomography-guided collection drainage (abdominal drain had been removed 3 days earlier)	Yes (15th POD)	13 days
7	28.56	Abdominal pain + bloating	No (Tazocin), already had a drain and ileostomy	No	13 days
8	20.61	Abdominal pain + abdominal distension + turbid drainage	Yes (EL - terminal colostomy - large amount of fecal contamination in cavity)	No	18 days
9	26.9	Abdominal pain + bloating + nausea + turbid drainage	No (was already drained and with ileostomy)	Yes (8th POD - due to ileostomy stoppage)	11 days
10	34.3	Bloating + tachycardia	Yes (EL - terminal ileostomy)	No	21 days
11	31	Abdominal pain + turbid drainage	Yes (EL - Hartmann)	No	7 days
12	22.6	Asymptomatic (had CT scan due to increased CRP)	Yes (4th POD - tomography-guided collection drainage; 5th POD - laparoscopy + protective ileostomy)	No	9 days
13	19.1	Asymptomatic (had CT scan due to increased CRP)	Yes (4th POD - Tazocin; 10th POD - Laparoscopy - Hartmann)	No	23 days
14	30.2	Abdominal pain + bloating + nausea	Yes (protective ileostomy)	No	13 days
15	18.2	Turbid drainage + tachycardia	Yes (protective ileostomy)	No	10 days
Patient	CRP on day 3 (mg/dL)	Clinical Symptoms	Surgical Procedures	Readmission	Length of hospital stay
16	34.1	Abdominal pain + bloating	Yes (3rd POD - EL - Hartmann; 8th POD - EL - enterotomy - ileal fistula)	No	33 days
17	45.1	Abdominal pain + abdominal distension + tachycardia	Yes (ileotransverse anastomosis reconstruction)	No	23 days
18	16.8	Bloating + turbid drainage	Yes (EL - Hartmann)	No	5th POD - death

**Graph 1 ch1:**
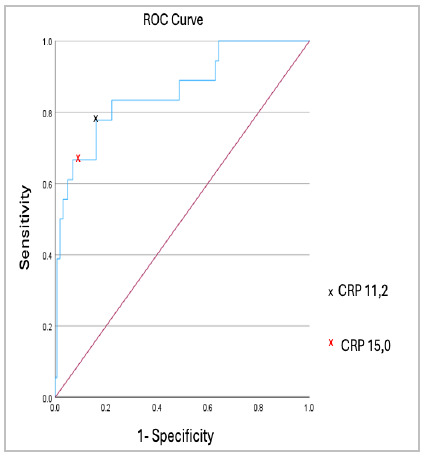
ROC Curve for C-Reactive Protein

The ROC curve analysis for CRP showed an area under the curve (AUC) of 0.860 (Graph 1). The cut-off value for the largest area under the curve was 11.2mg/dL, resulting in a sensitivity of 77.8% and a specificity of 84.0%. The negative predictive value (NPV) was 97.1%. Among all 18 cases of anastomotic dehiscence, only four patients had CRP levels below 11.2mg/dL. An alternative value with greater accuracy was 15.0mg/dL, with sensitivity and specificity of 66.7% and 90.7%, respectively. The NPV of 96.1% was very similar to that of the cut-off value of 11.2mg/dL, but the accuracy was higher for the value of 15mg/dL, with 88.3% compared to 83.3% for 11.2mg/dL (Table 3).

**Table 3:** Diagnostic measures of PCR value.

PCR (value)	Sensitivity	Specificity	PPV	NPV	Accuracy
11.2mg/dL	0.778	0.840	0.350	0.971	0.833
15.0mg/dL	0.667	0.907	0.444	0.961	0.883

[Fig ch1]

The area under the curve (AUC) is 0.86, showing a good predictive capacity of CRP as an inflammatory marker in the clinical diagnosis. The CRP point of 11.2 mg/L (black X) shows a sensitivity of 77.8% and a specificity of 84.0%. The CRP point of 15.0 mg/L (red X) indicates a sensitivity of 66.7% and a specificity of 90.7.

## DISCUSSION

Elevation in serum CRP levels after colorectal surgery is strongly associated with anastomotic leakage^6^. Our data demonstrated that CRP levels above 15mg/dL are related to a higher incidence of this complication. On the other hand, levels below this limit allowed a safe hospital discharge in our analysis if there were no clinical signs suggesting surgical complications, such as abdominal pain, abdominal distension, fever, tachycardia, or change in the appearance of abdominal drain contents.

In our study, we evaluated the relationship between CRP levels on the third postoperative day and the absence of colorectal fistula. Of the 180 colorectal surgeries performed, 18 had fistulas, only four of these patients had CRP levels below 11.2mg/dL, and six had CRP levels below 15mg/dL.

CRP kinetics peak on the second day after surgery, followed by a rapid decrease in uncomplicated patients. However, in the presence of a fistula, CRP levels remain elevated or increase^13^. Lagoutte et al., in a prospective study, and Warschkow et al., in a large retrospective series, suggested that a CRP level of 12.5mg/dL on the fourth postoperative day indicates a high risk of septic complications^14^ ^,^ ^15^. We found that a single CRP measurement on the third postoperative day could predict the presence of surgical complications, such as anastomotic leakage. After analyzing the ROC curve, we identified 11.2mg/dL as the ideal cutoff point. However, when comparing this value with a limit of 15.0mg/dL, the difference in negative predictive value and precision was negligible or even smaller, as seen in the case of accuracy. Therefore, to optimize early discharge, we adopted a threshold value of 15.0mg/dL.

Institutional protocol for early recovery aims to discharge patients between 3^rd^ and 5^th^ days after surgical procedures if the postoperative period is uneventful. In this protocol, CRP levels are evaluated on the 3^rd^ postoperative day. Patients with CRP below 15mg/dL and without clinical signs or complications, such as abdominal pain, tachycardia, fever, abdominal distension, or alteration in the appearance of drain contents, are considered fit to be safely discharged. On the other hand, if CRP is above 15mg/dL, surgical complications are immediately investigated by means of computed tomography, even in the absence of clinical signs of fistula. Patients with clinical alterations suggestive of surgical complications are promptly investigated, regardless of CRP value^16^. 

Matthiessen et al., in a study with 33 patients who underwent anterior rectum resection, 32 of whom were due to carcinoma, considered the period between the 3^rd^ and 8^th^ postoperative days as crucial for the diagnosis of fistula. They observed that the increase in CRP occurred from the second postoperative day, and the median number of days for clinical diagnosis was eight days^8^. Almeida et al. studied 173 patients undergoing colectomy procedures and identified a cut-off value of 140mg/L (14mg/dL) of CRP on the 3^rd^ postoperative day optimizes sensitivity and specificity for identifying the presence of a fistula^17^.

Of the 18 fistula cases we found, only six had a CRP value below 15mg/dL. However, they had at least one symptom associated with surgical complications such as abdominal pain, abdominal distension, or tachycardia, warranting further investigation. In two cases, no symptoms were presented, but the CRP levels were above the cut-off value, leading to an investigation that diagnosed fistula. In one case, the CRP value was 11.44mg/dL, but the patient had protective ileostomy and no intervention was necessary. This patient was diagnosed with an 8mm fistula 27 days later during an endoscopy examination in preparation for ileostomy closure. This demonstrates that, in asymptomatic patients, the CRP assessment on the 3^rd^ day below 15mg/dL was safe for hospital discharge and that in symptomatic patients, regardless of CRP value, appropriate investigation should follow.

Early identification of anastomotic leaks is crucial to reduce postoperative morbidity and mortality in cancer patients, as this complication is associated with higher rates of cancer recurrence and shorter disease-free survival^10^. The incorporation of CRP measurement into the early recovery protocol for colorectal surgeries allows rapid diagnosis of fistulas, reducing morbidity, reducing health care costs, shortening length of hospital stay, and ensuring safer discharges^18^ ^,^ ^19^.

The main limitation of this study is its observational design, which brings potential selection biases inherent to this type of methodology. However, it demonstrates the safety of the strategy adopted, considering the large surgical volume and the low complication rate. In addition, no patient was neglected as to complications, either due to early discharge or to CRP evaluation on the third day, reinforcing the safety of the strategy presented.

## CONCLUSION

Routine measurement of CRP below 15.0mg/dL on the 3rd postoperative day associated with clinical evaluation in asymptomatic patients proved to be effective in predicting a low risk of anastomotic dehiscence. This finding ensures safer early discharge and lower health care costs. On the other hand, CRP above 15mg/dL on the 3^rd^ postoperative day, regardless of symptoms, should promptly indicate an active search for a surgical complication.

## References

[1] Tsalikidis C, Mitsala A, Mentonis VI, Romanidis K, Pappas-Gogos G (2023). Predictive Factors for Anastomotic Leakage Following Colorectal Cancer Surgery Where Are We and Where Are We Going?. Curr Oncol.

[2] Jin D, Chen L (2021). Early prediction of anastomotic leakage after laparoscopic rectal surgery using creactive protein. Medicine (Baltimore).

[3] Phitayakorn R, Delaney CP, Reynolds HL, Champagne BJ (2008). Standardized Algorithms for Management of Anastomotic Leaks and Related Abdominal and Pelvic Abscesses After Colorectal Surgery. World J Surg.

[4] Branagan G, Finnis D (2005). Prognosis After Anastomotic Leakage in Colorectal Surgery. Dis Colon Rectum.

[5] Benoit O, Faron M, Margot N, Creavin B, Debove C, Tiret E (2019). C-Reactive Protein Values After Colorectal Resection Can We Discharge a Patient With a C-Reactive Protein Value &gt;100? A Retrospective Cohort Study. Dis Colon Rectum.

[6] Law WL, Choi HK, Lee YM (2007). Anastomotic Leakage is Associated with Poor Long-Term Outcome in Patients After Curative Colorectal Resection for Malignancy. J Gastrointest Surg.

[7] Smith SR, Pockney P, Holmes R, Doig F, Attia J, Holliday E (2018). Biomarkers and anastomotic leakage in colorectal surgery C-reactive protein trajectory is the gold standard. ANZ J Surg.

[8] Matthiessen P, Henriksson M, Hallböök O, Grunditz E, Norén B, Arbman G (2008). Increase of serum C-reactive protein is an early indicator of subsequent symptomatic anastomotic leakage after anterior resection. Colorectal Dis.

[9] Giaccaglia V, Salvi PF, Antonelli MS, Nigri G (2016). Procalcitonin Reveals Early Dehiscence in Colorectal Surgery. Ann Surg.

[10] Gray M, Marland JRK, Murray AF, Argyle DJ, Potter MA (2021). Predictive and Diagnostic Biomarkers of Anastomotic Leakage A Precision Medicine Approach for Colorectal Cancer Patients. J Pers Med.

[11] Degiuli M, Elmore U, de Luca R, de Nardi P, Tomatis M (2022). Risk factors for anastomotic leakage after anterior resection for rectal cancer (RALAR study) A nationwide retrospective study of the Italian Society of Surgical Oncology Colorectal Cancer Network Collaborative Group. Colorectal Dis.

[12] Singh PP, Zeng ISL, Srinivasa S, Lemanu DP, Connolly AB, Hill AG (2014). Systematic review and meta-analysis of use of serum C-reactive protein levels to predict anastomotic leak after colorectal surgery. Br J Surg.

[13] Ortega-Deballon P, Radais F, Facy O, d'Athis P, Masson D (2010). C-Reactive Protein Is an Early Predictor of Septic Complications After Elective Colorectal Surgery. World J Surg.

[14] Lagoutte N, Facy O, Ravoire A, Chalumeau C, Jonval L, Rat P (2012). C-reactive protein and procalcitonin for the early detection of anastomotic leakage after elective colorectal surgery pilot study in 100 patients. J Visc Surg.

[15] Warschkow R, Tarantino I, Torzewski M, Naf F, Lange J, Steffen T (2012). Diagnostic accuracy of C-reactive protein and white blood cell counts in the early detection of inflammatory complications after open resection of colorectal cancer a retrospective study of 1,187 patients. Int J Colorectal Dis.

[16] Doeksen A, Tanis PJ, Vrouenraets BC, Lanschot van JJ, Tets van WF (2007). Factors determining delay in relaparotomy for anastomotic leakage after colorectal resection. World J Gastroenterol.

[17] Almeida AB, Faria G, Moreira H, Pinto-de-Sousa J, Correia-da-Silva P, Maia JC (2012). Elevated serum C-reactive protein as a predictive factor for anastomotic leakage in colorectal surgery. Int J Surg.

[18] Muñoz JL, Alvarez MO, Cuquerella V, Miranda E, Picó C, Flores R (2018). Procalcitonin and C-reactive protein as early markers of anastomotic leak after laparoscopic colorectal surgery within an enhanced recovery after surgery (ERAS) program. Surg Endosc.

[19] Gozalichvili D, Binquet C, Boisson C, Guiraud A, Facy O, Ortega-Deballon P (2023). Early detection of anastomotic leak with C-reactive protein increases the chances of anastomotic salvage. Colorectal Dis.

[20] Oken MM, Creech RH, Tormey DC (1982). Toxicity and response criteria of the Eastern Cooperative Oncology Group. Am J Clin Oncol.

[21] Hortobagyi GN, Connolly JL, D'Orsi CJ, Amin MB, Edge S, Greene F, American Joint Committee on Cancer (2017). AJCC cancer staging manual.

